# Validation of the Psychological Insight Scale: A new scale to assess psychological insight following a psychedelic experience

**DOI:** 10.1177/02698811211066709

**Published:** 2022-01-05

**Authors:** Joseph M Peill, Katie E Trinci, Hannes Kettner, Lea J Mertens, Leor Roseman, Christopher Timmermann, Fernando E Rosas, Taylor Lyons, Robin L Carhart-Harris

**Affiliations:** 1 Department of Brain Sciences, Centre for Psychedelic Research, Imperial College London, London, UK; 2 Clinical Psychopharmacology Unit, Division of Psychology and Language Sciences, University College London, London, UK; 3 Data Science Institute, Imperial College London, London, UK; 4 Centre for Complexity Science, Imperial College London, London, UK; 5 Psychedelics Division, Neuroscape, University of California San Francisco, San Francisco, CA, USA

**Keywords:** Psychedelic, insight, emotion, mediation, well-being, therapy

## Abstract

**Introduction:**

As their name suggests, ‘psychedelic’ (mind-revealing) compounds are thought to catalyse processes of psychological insight; however, few satisfactory scales exist to sample this. This study sought to develop a new scale to measure psychological insight *after* a psychedelic experience: the Psychological Insight Scale (PIS).

**Methods:**

The PIS is a six- to seven-item questionnaire that enquires about psychological insight *after* a psychedelic experience (PIS-6) and accompanied behavioural changes (PIS item 7). In total, 886 participants took part in a study in which the PIS and other questionnaires were completed in a prospective fashion in relation to a planned psychedelic experience. For validation purposes, data from 279 participants were analysed from a non-specific ‘global psychedelic survey’ study.

**Results:**

Principal components analysis of PIS scores revealed a principal component explaining 73.57% of the variance, which displayed high internal consistency at multiple timepoints throughout the study (average Cronbach’s α = 0.94). Criterion validity was confirmed using the global psychedelic survey study, and convergent validity was confirmed via the Therapeutic-Realizations Scale. Furthermore, PIS scores significantly mediated the relationship between emotional breakthrough and long-term well-being.

**Conclusion:**

The PIS is complementary to current subjective measures used in psychedelic studies, most of which are completed in relation to the acute experience. Insight – as measured by the PIS – was found to be a key mediator of long-term psychological outcomes following a psychedelic experience. Future research may investigate how insight varies throughout a psychedelic process, its underlying neurobiology and how it impacts behaviour and mental health.

## Introduction

A growing body of evidence supports the potential utility of psychedelic compounds for aiding the treatment of numerous psychiatric disorders (for reviews, see Aday et al., 2020; Andersen et al., 2021; Carhart-Harris and Goodwin, 2017). Although the therapeutic promise of psychedelics is receiving growing empirical support, the mechanisms through which psychological outcomes occur are less well understood (Carhart-Harris, 2019; Nutt et al., 2020). Research demonstrates that ‘peak experiences’ (Maslow, 1964; Roseman et al., 2017) – including ‘emotional breakthrough’ – may be important mediators of long-term psychological changes after psychedelic use (Roseman et al., 2019). Using a generic altered state of consciousness scale, our team previously found scores on an ‘Insight’ subscale predicting subsequent improvements in depressive symptoms in a trial of psilocybin therapy for treatment-resistant depression (Carhart-Harris et al., 2018). In separate studies, greater self-reported insight was found in association with reduced alcohol/tobacco consumption after psychedelic use (Garcia-Romeu et al., 2019b; Noorani et al., 2018). Furthermore, acute insightfulness in association with a psychedelic experience was found to promote psychological flexibility and subsequent mental health improvements in individuals reporting histories of depression and anxiety (Davis et al., 2020a, 2020b, 2021b). Moreover, acute insight has been demonstrated to correlate with therapeutic outcomes such as decreased depressive symptoms (Davis et al., 2021a; Williams et al., 2021), anxiety symptoms and stress symptoms (Williams et al., 2021). Many studies have suggested that insight is a common, if not fundamental, property of the psychedelic experience (Carbonaro et al., 2020; Carhart-Harris and Nutt, 2010; Davis et al., 2020b, 2021a; Garcia-Romeu et al., 2019a; Lea et al., 2020; Savage et al., 1966; Sellers et al., 2018). Indeed, the word ‘psychedelic’ itself means ‘revealing the psyche or soul’ (Huxley, 1999), implicitly referencing insight-promotion as a core drug property.

### Measuring therapeutic-relevant aspects of the psychedelic experience

It is a reliable principle that the nature and quality of the acute psychedelic experience mediate long-term psychological changes post-experience (Roseman et al., 2017). Indeed, evidence suggests that ‘peak experiences’, ‘challenging experiences’ and ‘emotional breakthroughs’ are particularly pertinent aspects of the acute experience, bearing relevance to subsequent long-term psychological changes (Roseman et al., 2019). Psychedelic peak experiences have been described as being among the most personally meaningful events of one’s life (Griffiths et al., 2018), and evidence suggests that they may mediate enduring positive psychological effects following psychedelic use (for a review, see Barrett and Griffiths, 2018). Psychedelic peak experiences have been likened to ‘quantum change experiences’ (Griffiths et al., 2018) and ‘pivotal mental states’ (Brouwer & Carhart-Harris, 2021). The former are described as sudden and often profoundly meaningful experiences that result in dramatic and enduring positive behavioural, emotional and cognitive changes (Miller, 2004), whereas the latter are defined as hyperplastic states in which the likelihood of psychological transformation is greatly enhanced.

The Mystical Experience Questionnaire (MEQ; MacLean et al., 2012), originally derived from the States of Consciousness Questionnaire (SOCQ; Griffiths et al., 2006; Pahnke, 1963), was devised to capture specific qualities of the psychedelic peak experience, including subscales related to ‘Mystical’, ‘Transcendence of Time and Space’, ‘Positive Mood’ and ‘Ineffability’ (MacLean et al., 2012). Consistently, the psychedelic peak experience – as measured using the MEQ and related dimensions of the 5- and 11-dimensional Altered States of Consciousness Questionnaire (5D-ASC, Dittrich et al., 2010; 11D-ASC, Studerus et al., 2010) – has been associated with enduring positive outcomes in both healthy groups and patient populations (Davis et al., 2020a; Garcia-Romeu et al., 2019b; Griffiths et al., 2008; Roseman et al., 2019).

Psychologically challenging experiences, characterised by psychological struggle or distress, have been reported in various psychedelic studies (Barrett et al., 2016; Carbonaro et al., 2016; Haijen et al., 2018) and are linked to the vernacular term ‘bad trip’. To capture this phenomenon, the Challenging Experience Questionnaire (CEQ) was developed (Barrett et al., 2016). Studies have reported mixed results for the relationship between challenging experiences and long-term psychological outcome (Barrett et al., 2016; Carbonaro et al., 2016; Haijen et al., 2018). One study found challenging psychedelic experiences to be associated with subsequent positive outcomes, but longer duration challenging experiences were predictive of negative outcomes (Carbonaro et al., 2016). Inconsistent with previous work (Carbonaro et al., 2016), a separate prospective survey study of ours found that CEQ scores did not predict subsequent changes in psychological well-being (Haijen et al., 2018).

It is a fundamental principle of psychodynamic psychology that a surrender or ‘letting go’ of psychological resistance is required for the occurrence of emotional breakthrough, ‘abreactions’ or ‘catharses’ (Crocket et al., 1963; Eisner and Cohen, 1958; Fisher, 2015; Freud, 1920; Grof, 1980). This notion is supported by patient references to emotional breakthroughs in post-psychedelic treatment reports (Belser et al., 2017; Gasser et al., 2015; Watts et al., 2017). The development of the Emotional Breakthrough Inventory (EBI; Roseman et al., 2019) was inspired by a desire to address this phenomenon via a focused quantitative scale. A recent factor analysis confirmed the EBI to be sufficiently orthogonal to the MEQ and CEQ and able to predict long-term changes in well-being post-experience (Roseman et al., 2019).

### Insight

Developing insight is a key aim and fundamental component of many psychotherapies (Jennissen et al., 2018) and appears to play an important role in developing self-growth and self-awareness (Noorani et al., 2018). Within psychodynamic theory, greater self-understanding is thought to result in an enhanced capacity to respond to stresses and negative experiences adaptively (Altman, 1964; Rosenblatt, 2004), promoting greater well-being and life satisfaction (Harrington and Loffredo, 2011; Lyke, 2009). Furthermore, a chief component of cognitive–behavioural therapy (CBT) is developing insights into one’s negative patterns of thought (Grosse Holtforth et al., 2007). A meta-analysis on psychodynamic, CBT and counselling found a moderate association between insight and psychotherapy outcome (Jennissen et al., 2018). Gaining insight into one’s thoughts, behaviours and experiences is thought to help reduce symptoms by enabling individuals to first understand their difficulties, reduce distorted negative beliefs and, eventually act on, and master these difficulties through conscious cognitive and behavioural changes (Lacewing, 2014; Rosenblatt, 2004). Moreover, cultivating insight is a key factor in some forms of meditation; specifically, Vipassanā – a form of meditation that is sometimes translated as ‘insight’ or more accurately ‘special-seeing’. Vipassanā meditation has been found to foster improvements in emotional processing (Wu et al., 2019) and well-being (Krygier et al., 2013) in healthy individuals. Taken together, these findings demonstrate that developing personal insight into one’s own thoughts, feelings and behaviours is conducive to mental well-being.

Psychological insight has been measured using the Insight subscale of the self-reflection and insight scale (SRIS-IN; Grant et al., 2002). The authors define insight as ‘the clarity of understanding of one’s thoughts, feelings and behavior’. The phrasing of this scale’s items does not allow for an assessment of changes in insight in relation to a time-limited experience (e.g. a psychedelic experience). Hence, this scale is not well suited to assessing whether an individual has gained psychological insights following a psychedelic experience, or indeed any other time-limited, but potentially transformative experience. The less commonly used Therapeutic-Realizations Scale (TRS) was designed for use in psychotherapy to assess the beneficial effects (such as gaining insight, feeling encouragement, clarifying a problem) at the session level (Kolden, 1991, 1996; Kolden et al., 2000). The TRS is comprised of three subscales, most pertinent to the present study is the Mastery of Insight Scale (MIS). The MIS aims to capture ‘mastery’, that is, the perception that one is able to cope and function well, and ‘insight’, that is, having greater understanding of one’s present behaviours and feelings. While this subscale relates to changes in insight with respect to an experience, the MIS does not address how past experiences may influence present thoughts, feelings and behaviours (Kolden et al., 2000).

In the context of psychedelic experiences, a Psychological Insight Questionnaire (PIQ) was recently devised (Davis et al., 2020a, 2021b). The authors define insight as ‘realizations or discoveries about personality, relationships, behavioral patterns or emotions’. Within the PIQ, participants are asked to report on the intensity of the items that they may have experienced during the session. Therefore, the PIQ appears to be designed for sampling insights gained *during* a psychedelic session rather than post-acutely. Currently, a scale does not exist that aims to determine particular insights that have developed during the days and weeks following a psychedelic experience. Previous research has demonstrated that this subacute period after a psychedelic experience, sometimes referred to as the ‘after glow’, is important for developing self-reflection and integration of one’s thoughts and feelings (Lepore and Helgeson, 1998; Roseman et al., 2019; Ross et al., 2016). We have therefore chosen to develop a scale – Psychological Insight Scale (PIS) – that assesses insight crystallising *after* a psychedelic experience. Moreover, we predict that its greatest value may be as a subacute predictor or mediator of long-term mental health outcomes, where high PIS scores predict greater and more sustained improvements.

A consistent theme within current definitions of insight follows the idea of ‘novel’ thoughts or realisations. For the purpose of the present study, we adopt the following definition of insight: *the coming upon of a new perspective on one’s self or life*. Thus, the PIS assesses *personal* psychological insight and, unlike other scales (e.g. the MEQ), is not specifically designed to assess transpersonal or philosophical insight, for example, pertaining to metaphysical beliefs.

Given the limitations of currently available questionnaires to test psychological insight following psychedelic experiences, the aim of the present study was to psychometrically validate a novel PIS. Factor structure and internal reliability, as well as criterion (i.e. pre- and postdictive validity) of the PIS were tested in a prospectively assessed sample of psychedelic retreat participants. Concurrent validity of the PIS was assessed via its correlation with psychological well-being. In addition, the PIS was externally validated in a second prospective sample of individual psychedelic users taking psychedelics in a broad range of contexts. Convergent validity of the PIS was investigated by comparing its scores with those from the MIS and the SRIS-IN. As with many insight-related measures (Gori et al., 2015; Grant et al., 2002; Kolden, 1991), the insight explored in the present study is subjective in nature and is not externally validated.

Our main hypotheses were as follows:

*H1 (Predictive Validity)*: PIS scores at 1-day post-retreat will be predictive of enhanced well-being reported 2 weeks post-retreat.
*H2 (Concurrent Validity)*: PIS scores will be positively correlated with well-being scores, both reported 2 weeks post-retreat.
*H3 (Postdictive Validity)*: The acute psychedelic experience (assessed via EBI, MEQ and CEQ) will significantly predict post-acute psychological insight.
*H4*: PIS scores at +1 day will significantly mediate the relationship between acute emotional breakthrough and mystical experience and subsequent increases in well-being.
*H5 (Convergent Validity)*: PIS scores will be convergent with MIS and SRIS-IN scores.

## Methods

### Ethics

These studies were approved by the Joint Research Compliance Office and the Imperial College Research Ethics Committee (ICREC; study 1 ICREC reference 18IC4346, study 2 ICREC reference 17IC3746). All participants gave written informed consent before admission to the study. This was an observational study, and hence all psychedelic drugs used were taken by individual’s accord.

### Psychometric measures

Only those measures relevant for the current study are described below.

#### EBI

A six-item questionnaire introduced in Roseman et al. (2019) to assess acute emotional breakthroughs, based on the Visual Analogue Scale (VAS) scoring system, where 0 is ‘No, not more than usual’ and 100 is ‘Yes, entirely or completely’. The EBI score has been shown to correlate with long-term well-being (2 weeks), indicating its predictive validity. In addition, the EBI has demonstrated high internal consistency (Cronbach’s α = 0.932).

#### MEQ

The revised 30-item MEQ, which assesses the acute mystical-type experience via a 5-point Likert-type scale, grouped into 4 subscales: ‘Mystical’, ‘Positive Mood’, ‘Transcendence of Time and Space’ and ‘Ineffability’ (MacLean et al., 2011). This questionnaire has demonstrated high internal consistency (Cronbach’s α = 0.933). Moreover, various studies have demonstrated strong predictive validity of the MEQ with respect to long-term psychological measures such as personal meaning (Garcia-Romeu et al., 2019b; Griffiths et al., 2008). A recent validation of the MEQ demonstrated its high reliability and validity within controlled psilocybin studies (Barrett et al., 2015).

#### CEQ

Developed by Barrett et al. (2016), the CEQ was created to analyse challenging events during a psychedelic experience. The CEQ is a 26-item, 5-point Likert-type scale with seven subscales investigating Fear, Paranoia, Insanity, Physical Distress, Isolation, Death and Grief. Internal consistency for the subscales ranged (Cronbach’s α = 0.65–0.89). Overall internal validity was later reported as excellent for the total scale (Cronbach’s α = 0.95; Davis et al., 2021b).

#### The Warwick–Edinburgh Mental Well-Being Scale

The Warwick–Edinburgh Mental Well-Being Scale (WEMWBS) is a validated, 14-item scale that was developed to assess positive mental health, which captures both hedonic and eudaimonic perspectives of psychological functioning (Tennant et al., 2007). Responses are gauged using a 5-point Likert-type scale where 1 to 5 are ‘none of the time’, ‘rarely’, ‘some of the time’, ‘often’, ‘all the time’, respectively. In addition to satisfaction in interpersonal relationships, the WEMWBS also takes into account a range of aspects of positive affect and functioning. Hence, this scale can be used to investigate the improvement or change in well-being within subject and at a group level (Maheswaran et al., 2012). The WEMWBS demonstrates a high internal consistency (Cronbach’s α = 0.91; Tennant et al., 2007).

### Construction of the PIS

The PIS was developed and revised by a group of experts within the Centre for Psychedelic Research at Imperial College London (Dr Robin Carhart-Harris, Hannes Kettner, Dr Taylor Lyons, Dr Leor Roseman and Dr Christopher Timmermann). The items within the PIS were devised after distilling observations of patient-reported ‘insight’ across multiple psychedelic studies (please see ‘Participant Reports’ section in the ‘Results’ section).

The PIS was designed to assess changes in individuals’ level of psychological insight following a psychedelic experience (or other treatment/experience) compared with their baseline state (i.e. before the experience). The PIS is a seven-item scale comprised of two components: a core psychological component (six items) that captures various aspects of insight, and a supplementary behavioural component (one item) that seeks to capture positive behavioural changes which were motivated by/associated with the newly gained insights. The two components will be referred to as PIS-6 and PIS item 7, respectively, and analysed separately. The scale can be utilised to assess insights occurring at any given timepoint following a psychedelic experience, therapeutic session or other event, and to assess any accompanied behavioural change. Answers are given using a VAS scoring system (see appendices of Supplemental Material).

### Online prospective studies: Study 1

Recruitment of participants occurred via online advertisements as well as through psychedelic retreat providers. Retreat centres invited their prospective clients to take part in this scientific study by visiting the study page (www.ceremonystudy.com), which contained participant information and the opportunity to sign informed consent. Following this, participants received a series of email reminders at various timepoints before and after their indicated retreat date.

Data were collected at the following timepoints: T1 – Baseline (2 weeks before the ceremony), T2 – post-experience (1 day following a psychedelic ceremony), T3 – post-retreat (1 day after the end of the retreat) and T4 – endpoint 1 and T5-endpoint 2 (2- and 4 weeks after the retreat ended, respectively). The following data were requested at each timepoint: T1 demographics and baseline scores of well-being (WEMWBS), T2 acute subjective experience measures (MEQ, EBI, CEQ), T3 post-acute measures (PIS), and T4 and T5 psychological outcomes (PIS, WEMWBS). In addition to the structured questionnaires, participants also could report freely on their psychedelic experience. Selected accounts are included in the present article to highlight participants that chose to report on ‘Insight’.

#### Principal components analysis

To determine the interrelationships between individual items within the PIS-6, the principal components analysis (PCA) was implemented. Before performing PCA, Kaiser–Meyer–Olkin (KMO) and Bartlett’s tests were utilised to determine the method’s adequacy. The number of factors was determined by standard methods relying on scree plot criteria, as described by Cattell (1966). The internal consistency of the questionnaire was assessed using Cronbach’s alpha (Cronbach, 1951). PIS item 7 was excluded from the PCA as this item reflects a behavioural component and as such does not directly seek to assess psychological insight.

#### Criterion validity (postdictive and predictive validity)

Path analysis (Pearl, 2012) was performed on the ceremony survey dataset to test the predictive and postdictive validity of the PIS-6. These analyses included the following variables: (1) mystical-type (MEQ), emotional breakthrough (EBI) and challenging experiences (CEQ) as indicators of acute psychedelic effects, measured on the day after ceremony predicting; (2) psychological insight (PIS-6) measured on the day after end of the retreat, which in turn was used to predict; and (3) psychological well-being (WEMWBS) at the first endpoint 2 weeks after the retreat, while controlling for baseline well-being scores.

Following recommendations put forward by (Kline, 2015) several indicators of overall fit are reported, including the model chi-square, root mean square error of approximation (RMSEA), comparative fit index (CFI) and standardised root mean square residual (SRMR). Direct effects of CEQ, MEQ and EBI on WEMWBS at 2 weeks post-ceremony were included, as well as indirect effects mediated through psychological insight (PIS-6). Total effects were calculated by summing direct and indirect effects on well-being for MEQ, CEQ and EBI, respectively. Maximum likelihood parameter estimation was chosen as the estimation method since all included data were continuous; 1000 bootstrap samples were drawn to infer confidence intervals (CIs) for parameter estimates (*B*) and determine significance of indirect effects. Following Acock (2014), effect strengths were interpreted based on standardised beta coefficients, with β < 0.2 being interpreted as weak, 0.2 < β < 0.5 as moderate and β > 0.5 as strong effects.

#### Concurrent validity

To analyse the relationship between well-being and psychological insight at 2 weeks, correlation analyses between the PIS and WEMWBS were implemented. For timepoint T3, Spearman’s correlation coefficient was utilised to assess concurrent validity between PIS (PIS-6 and PIS item 7 separately) and well-being (ΔWEMWBS = WEMWBS_+2 weeks_ – WEMWBS_Baseline_).

#### Predictive validity in subsample with low-well-being

Spearman’s rank correlation was performed on PIS 1 day post-retreat and ΔWEMWBS (2 weeks) in a low well-being subsample to explore whether PIS-6 is a stronger predictor of changes in well-being for these individuals. Similar to previous studies (Santini et al., 2020; Stewart-Brown et al., 2015), the low well-being subpopulation was defined as WEMWBS score of ⩽43 at baseline.

### Study 2: External and convergent validation

To examine whether findings from within a ceremony setting hold true in other settings and contexts, external validity was assessed using data from a second prospective online survey (www.global.psychedelicsurvey.com). This second survey was completed by individuals taking a psychedelic drug in a range of different contexts; we called this the ‘global psychedelic survey’. To sign up for the survey, respondents provided informed consent and confirmed they were aged 18+. In addition, the inclusion of insight-related psychometric scales in this survey also allowed verification of convergent validity. In the following text, details about the external and convergent validation are discussed.

#### External validity

The previously detailed measures used in the ceremony survey were also employed in study 2, but the timepoints differed slightly: in the latter, the measures were completed with respect to a single psychedelic experience, as opposed to the ceremony data where measures sometimes corresponded to multiple psychedelic experiences (as part of a single retreat). In study 2, the PIS was first completed 1-day post-experience, at the same time of completion as the acute measures (MEQ, EBI and CEQ). Spearman’s rank correlation analyses were employed to assess predictive validity PIS-6 +1-day post-experience and change in well-being after 2 weeks (ΔWEMWBS = WEMWBS_+2 weeks_ – WEMWBS_Baseline_) for the total sample and a subsample with low well-being (baseline WEMWBS score ⩽43). In line with study 1 analyses, concurrent validity on PIS-6 and PIS item 7 were assessed via Spearman’s rank correlation analyses with change in well-being 2 weeks post-experience (ΔWEMWBS). Finally, to confirm postdictive validity, a multiple linear regression model was utilised to examine the predictive power of acute EBI, MEQ and CEQ on PIS at 2 weeks post-experience.

#### Convergent validity

To further assess construct validity of the PIS, convergent validity was analysed by comparison of PIS-6 scores with scores on existing measures of insight, which are described below. Note, these measures were not employed in study 1.

#### SRIS

The SRIS is comprised of 20 items with responses gauged via a 6-point Likert-type scale from 1 = ‘strongly disagree’ to 6 = ‘strongly agree’ (Grant et al., 2002). Two subscales make up the SRIS, namely, ‘Insight’ (SRIS-IN, consisting of 8 items) and ‘Self-Reflection’ (SRIS-SR, consisting of 12 items). The SRIS-IN measures the presence of insight into one’s self, and is positively correlated with psychological flexibility and negatively correlated with depression (Grant et al., 2002), demonstrating convergent validity. Cronbach’s alpha coefficients were 0.91 and 0.87 for the SRIS-SR and SRIS-IN, respectively.

#### MIS

Due to the lack of psychometric scales assessing *changes* in insight with respect to an ‘event’/experience, and in order to further explore convergent validity, the MIS from the validated TRS was used in isolation (Kolden, 1991, 1996). The TRS assesses the beneficial impacts (e.g. gaining understanding, feeling encouraged) of psychotherapeutic sessions and has a high internal consistency of 0.86 (Cronbach’s α). The MIS subscale is comprised of five items, which specifically assess session impacts and changes in aspects of self-efficacy and psychological insight. The items of MIS were scored on a 3-point Likert-type scale, where 0 = ‘not at all’, 1 = ‘moderate’ and 2 = ‘’a lot’.

Spearman’s rank correlation analyses were performed on PIS-6 and MIS at 1-day post-experience, and PIS-6 with SRIS-IN at 4 weeks post-experience.

### Statistical analyses

All the statistical analyses and graph formation were performed with SPSS V26 (IBM corporation, Armonk, NY, USA, 2017) and GraphPad Prism 8.0.2 (GraphPad Software, Inc., La Jolla, CA, USA).

### Data extraction and cleaning

All data were exported and prepared using MATLAB (release 2019b), deleting any incomplete responses (*n* = 160). Upon visual screening of two complete responses that had been flagged for data quality reasons (i.e. responses that continuously used the same or patterned response options, or were faster than 99% of respondents, indicative of an insufficient engagement with the survey items), the responses were recognised as valid, meaning that no cases had to be removed due to data quality issues.

## Results

### Study 1: Ceremony Survey

#### Participants

A total of 886 participants took part in study 1 (ceremony study). A questionnaire completion rate of 61.6% was recorded, with all other participants giving unanswered or partial responses to the questionnaires. For detailed information of demographics, please see Table 1.

**Table 1.** Demographics (Ceremony Study) (*N* = 886).

Total	*N* = 886
Age	
19–75	Mean = 44.4, SD = 12.6
Gender	
Male	455
Female	359
Other	5
NA	67
Nationality	
USA	359
GB	160
AU	31
DE	28
Other	241
NA	67
Education	
None	6
High school or equivalent (GED)	62
Associate/technical degree	58
College diploma	250
Master’s degree	275
Doctorate or professional degree	168
NA	67
Employment status	
Unemployed	60
Student part-time	13
Student full-time	33
Working part-time	120
Working full-time	520
Retired	73
NA	67
Previous psychedelic use	
No (psychedelic naïve)	330
Yes	489
NA	67
Psychedelic use within last 6 months	
None	569
1	83
2–5	129
6–10	23
11–20	10
21–50	5
50+	0
NA	67
Psychiatric illness	
No lifetime psychiatric diagnoses	539
At least one lifetime psychiatric diagnosis	280
NA	67

SD: standard deviation; GED: General Educational Development.

Demographic information was not available (NA) for 67 study participants who did not complete the baseline assessment.

#### Drug type

Participants stated what substance/substances were being taken during the ceremony. The following is a list of the drugs that were included within this dataset: psilocybin/magic mushrooms/truffles (74%), Ayahuasca (14%), N,N-dimethyltryptamine (DMT) (0.6%), lysergic acid diethylamide(LSD) or 1-propionyl-d-lysergic diethylamide (1P-LSD) (0.6%), San Pedro (0.6%), other (2.2%) and blank/unknown (8%).

#### Participant reports

In an open-ended question inviting respondents to describe details of their psychedelic ceremony experience, many subjects reported experiencing insight, with the term being explicitly mentioned in several reports. Below is a summary of selected accounts.

*At times I felt I gained powerful insights into myself and my behaviours.*

*Revealing deep insightful, accurate, poignant relief.*

*Coming up with good insights to questions that I had in my mind at that moment.*

*I had insights into my failure to love; that I had not reciprocated the near unconditional love from my sister. I felt an overwhelming sense of shame. I will henceforth love her unconditionally.*

*Mourning for the life I lived, thankful for the insight, for this ray of light in a pitch-black universe.*

*Meta-aware, insight, humility, group mind, save humanity.*

*Realisation. Understanding. Insight.*

*Later in the trip I was able to let go even more and speaking to one of the facilitators I was able to express an insight about the earliest years of my life.*

#### PCA

The first six items from the PIS were inputted into a PCA. A KMO test revealed a value of 0.855, demonstrating that 85.5% of the total variance is shared between the variables. The Bartlett’s test for sphericity was significant (χ^2^ = 1513.2, *p* < 0.001). Taken together, these values demonstrate that the variables have a suitable sampling adequacy and can be loaded onto factors, that is, they are suitable for PCA.

A total of 73.57% of the variance was explained by the first component (eigenvalue = 4.4), contrasting with only a <8.3% of variance being explained by the second factor (eigenvalue = 0.5). This points towards a unifactorial structure of the six-item PIS (Table 2), which was further confirmed via Cattell’s (1966) scree plot criterion.

**Table 2.** PCA of the PIS-6.

Item	Component 1
I have had important new insights about how past events have influenced my current mental health and behaviour	0.859
I have learned important new ways of thinking about my ‘self’ and my problems	0.872
I have had important new insights about how I would like to change aspects of myself or my lifestyle	0.881
I have become more conscious of aspects of my past that I used to ignore or not be fully aware of	0.849
I have become more conscious of aspects of my ‘self’ that I used to ignore or not be fully aware of	0.878
I have become more conscious of aspects of my lifestyle that I used to ignore or not be fully aware of	0.804

PCA: Principal components analysis; PIS: Psychological Insight Scale.

Loadings of first six items from the PIS fall into one single component. The seventh item has been omitted in this case due to the distinct differences in question phrasing. The seventh item incorporated into the PIS-7 reads: ‘I have made positive changes to my lifestyle and/or behaviour in accordance with the insights I have gained as part of my treatment/experience’.

#### Internal consistency

Evaluation of internal consistency for the PIS revealed excellent reliability for all timepoints (Cronbach’s α = 0.93, 0.94 and 0.95 for 1 day, 2 weeks and 4 weeks post-retreat, respectively).

#### PIS: Descriptive statistics

Following their experience(s), virtually all of the participants reported having gained some degree of insight at +1 day, +2 weeks and +4 weeks (i.e. >99% gave non-zero scores). PIS-6 scores were highest 1 day following the experience (mean = 63.8, SD = 27.9), with slight decreases after 2 (mean = 61.6, SD = 28.0) and 4 weeks (mean = 57.5, SD = 29.6). There was a moderate negative skewness of PIS scores across timepoints (–0.71, –0.64 and –0.44, respectively), and kurtosis = –0.57, –0.59 and –0.97 across respective timepoints. In respect to PIS item 7, scores were lowest at +1 day post-retreat (mean = 55.7 SD = 31.7), increasing to the highest at 2 weeks (mean = 58.6, SD = 30.2), before lowering slightly at 4 weeks (mean = 57.3, SD = 30.2). There was a moderate negative skewness of behavioural item scores across timepoints (–0.41, –0.50 and –0.40, respectively), and kurtosis = –1.0, –0.86 and –0.95 across respective timepoints.

#### Predictive and postdictive validity

**Figure 1.**
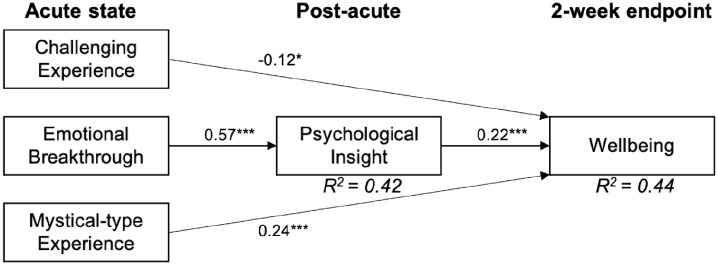
In a causal path analysis model, psychological well-being 2 weeks after a psychedelic experience is significantly predicted by the acute psychedelic state (CEQ, EBI, MEQ), while controlling for baseline well-being (not shown). Mystical and challenging experiences directly affect well-being, whereas the effect of emotional breakthrough experiences was mediated through psychological insight. Only paths and standardised coefficients significant below *p* = 0.05 are displayed. *p < 0.05, **p < 0.01, ***p < 0.001. CEQ: Challenging Experience Questionnaire; EBI: Emotional Breakthrough Inventory; MEQ: Mystical Experience Questionnaire.

The results of the path analysis are presented in Figure 1; standardised regression coefficients were displayed only if they were statistically significant. This model had near-perfect fit with a χ^2^ = 0.18 (df = 1, *p* = 0.67), RMSEA = 0, CFI = 1.0 and SRMR = 0.005 with a sample size of *n* in analysis = 283.

The model indicated a moderate direct effect of insight (PIS, β = 0.22, *p* < 0.001) on well-being 2 weeks after the psychedelic retreat. In addition, a significant, but weak, negative direct effect of challenging experiences (CEQ, β = –0.12, *p* < 0.05) and a moderate positive effect of mystical-type experiences (MEQ, β = 0.24, *p* < 0.001) on well-being were revealed. As expected, baseline well-being also exhibited a strong direct effect on well-being at the 2-week endpoint (β = 0.41, *p* < 0.001). The EBI showed a significant indirect effect on well-being (β = 0.13, *B* = 0.006, bootstrap CI = (0.003, 0.010), *p* < 0.001) that was mediated by insight (PIS) scores. The effect of emotional breakthrough on post-acute insight was the strongest relationship in the model (β = 0.58, *p* < 0.001).

*R*-square indices showed that baseline well-being, MEQ, CEQ, EBI and PIS-6 together accounted for approximately 44% of variance in well-being at the 2-week endpoint. In addition, MEQ, CEQ and EBI accounted for 42% of variance in post-retreat PIS-6. In the specified model, 57% of the total effect of EBI on well-being were mediated by insight, whereas only 8% of the effect of MEQ were indirect. Standardised parameter coefficients and significance of direct, indirect and total effects can be found in the appendices of Supplemental Material (Table 4).

#### Predictive validity within low well-being subgroup

**Figure 2.**
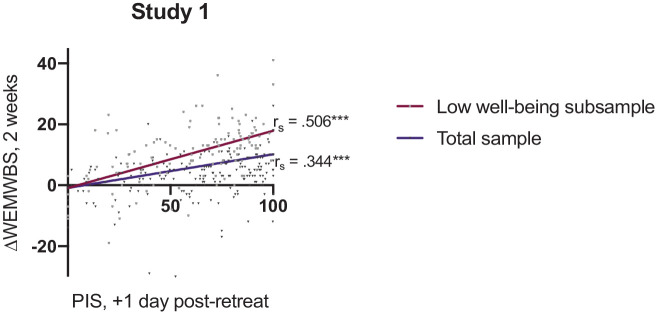
Correlation analysis between well-being scores (WEMWBS) at 2 weeks post-retreat and psychological insight (as measured using the PIS) at 1-day post-retreat. Participants with low baseline WEMWBS (⩽43) demonstrated the strongest correlation (*r_s_* = 0.506, *n* = 120), followed by all participants (*r_s_* = 0.344, *n* = 312). Displayed are the Spearman’s correlation coefficients (*r_s_*). WEMWBS: Warwick–Edinburgh Mental Well-Being Scale; PIS: Psychological Insight Scale. ***p < 0.001.

Participants within the low well-being subgroup (i.e. baseline WEMWBS score ⩽43) demonstrate a moderate–strong correlation (*r_s_* = 0.506, *p* < 0.001, *n* = 120) between PIS scores (+1-day post-retreat) and change in well-being at 2 weeks (ΔWEMWBS_2 weeks_) (Figure 2). For comparison, the total sample demonstrates a moderate correlation (*r_s_* = 0.344, *p* < 0.001, *n* = 312) between PIS (+1 day post-retreat) and change in well-being at 2 weeks.

#### Concurrent validity

Spearman’s rank correlation analysis revealed a moderate positive relationship of well-being change 2 weeks post-retreat with both psychological insight (*r_s_* = 0.434, *p* < 0.001, *n* = 416, one-tailed) and rating of reported behavioural changes (i.e. item 7) made in accordance with insight (*r_s_* = 0.415, *p* < 0.001, *n* = 392, one-tailed).

### Study 2: Global Psychedelic Survey

#### Participants and drug type

From the second survey (Global Psychedelic Survey), data were included from 279 individuals who participated in this survey (Table 3). A completion rate of 48.8% was recorded, with all other participants giving unanswered or partial responses to the questionnaires. Drug use included psilocybin (45%), LSD (41%), *N,N*-DMT (6%), Ayahuasca (3%), ketamine (2%), 5MEO-DMT (1%) and other (2%). Participants below the age of 18 (*n* = 8) were excluded from all analyses.

**Table 3.** Demographics (Global Psychedelic Survey) (*N* = 279).

Total	*N* = 279
Age	
18–69	mean = 30.9, SD = 10.7
Gender	
Male	162
Female	78
Other	6
NA	33
Ethnicity	
White	220
Black/African American	3
Asian	9
Other	14
NA	33
Education	
Left school before age 16 without qualifications	9
Some high school	17
High school diploma	39
Some university (or equivalent)	56
Bachelor’s degree (or equivalent)	64
Postgraduate degree (e.g. master’s or doctorate)	61
NA	33
Employment status	
Student	78
Full-time job	115
Part-time job	29
Unemployed	20
Retired	4
NA	33
Lifetime psychedelic use	
Yes	225
No (psychedelic naïve)	21
NA	33
Psychedelic use within the last 6 months	
None	46
Once	39
2–5 times	96
6–10 times	27
11–20 times	11
21–50 times	4
51–100 times	2
NA	54
Psychiatric illness	
No lifetime psychiatric diagnosis	155
At least one lifetime psychiatric diagnosis	91
NA	33

SD: Standard deviation.

Demographic information was not available (N/A) for 33/279 study participants who did not complete the baseline assessment.

#### External validity

##### Confirming construct validity

Similar to Ceremony Survey, PIS-6 scores demonstrated extremely high internal reliability (Cronbach’s α = 0.95) 1-day post-experience. In addition, psychological insight (PIS-6) and the behavioural changes made in accordance with insight (item 7) were strongly correlated at 2 weeks post-experience (*r_s_* = 0.683, *p* < 0.001, one-tailed, *n* = 221).

#### Descriptive statistics PIS

The majority of participants (>91%) reported gaining some degree of insight (i.e. non-zero scores) at each timepoint following their psychedelic experience: 1 day (mean = 47.7, SD = 32.7), 2 weeks (mean = 56.3, SD = 30.6) and 4 weeks (mean = 54.3, SD = 30.0) post-experience. PIS behavioural (item 7) scores showed a similar temporal pattern: 1 day post-experience (mean = 45.2, SD = 35.1), 2 weeks post-experience (mean = 52.0, SD = 31.9) and 4 weeks post-experience (mean = 54.56, SD = 31.87).

#### Predictive validity

**Figure 3.**
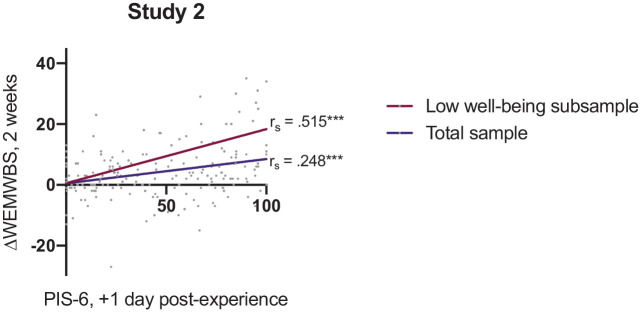
Predictive validity in study 2: Correlation analyses of psychological insight (PIS-6) measured 1-day post-psychedelic experience with changes in well-being (as measured by the WEMWBS) after 2 weeks (ΔWEMWBS_2 weeks_) within the total sample (*n* = 190) and low well-being subgroup (baseline WEMWBS score ⩽43, *n* = 69). Displayed are the Spearman’s correlation coefficients (*r_s_*). WEMWBS: Warwick–Edinburgh Mental Well-Being Scale. ***p < 0.001.

Spearman’s rank correlation analysis revealed that post-acute insight (PIS at +1 day) was positively associated with change in well-being 2 weeks post-experience (*r_s_* = 0.248, *p* < 0.001, *n* = 190; Figure 3) further confirming H1 – that is, that PIS scores soon after a psychedelic experience can predict subsequent long-term changes in well-being. As in study 1, this relationship was stronger within the low well-being subgroup (*r_s_* = 515, *p* < 0.001, *n* = 69; Figure 3).

#### Concurrent validity

In line with the results from the ceremony survey and H2 (i.e. PIS and WB will correlate in time), a moderate positive relationship between psychological insight and change in well-being 2 weeks post-experience was found by considering their Spearman’s rank correlation (*r_s_* = 0.271, *p* < 0.001, one-tailed, *n* = 198). Moreover, there was a moderate correlation between reported positive behavioural changes influenced by psychological insight (i.e. item 7) and change in well-being at 2 weeks post-experience (*r_s_* = 0.316, *p* < 0.001, *n* = 198).

#### Postdictive validity

A multivariate regression model was constructed with PIS at 2 weeks post-experience as the target variable, using acute measures of EBI, MEQ and CEQ as predictors. As with study 1, the results provide clear support for H3, with the model accounting for 40% of the variance of PIS (*R*^2^ adjusted = 0.405, *F*(3, 101) = 24.63, *p* < 0.001). In particular, psychological insight at 2 weeks post-experience exhibited significant associations with emotional breakthrough (β = 0.473, *p* < 0.001) and mystical experiences (β = 0.270, *p* = 0.007), but not with challenging experiences (β = –0.85, *p* = 0.300).

#### Convergent validity

A significant, albeit weak, positive correlation between PIS and SRIS-IN subscale (*r_s_* = 0.180, *p* = 0.008, one-tailed, *n* = 182) was found for the measures taken at 4 weeks post-experience. Such small correlation between SRIS-IN and PIS scores indicates that the convergence of these scales is weak. In line with H5, the MIS demonstrated high convergence with the PIS at +1 day post-experience (*r_s_* = 0.797, *p* < 0.001, one-tailed, *n* = 266), which suggests that the PIS samples a consistent phenomenon to that measured via the MIS.

## Discussion

The present work sought to introduce and validate the PIS, a novel scale designed to measure personal psychological insight *after* a psychedelic experience (PIS-6), and how such insight may translate into behavioural changes (PIS item 7). The present article examined the factor structure of the PIS-6 as well as its internal and external validity. In addition, we evaluated how insight – as measured using the PIS – mediates the relationship between elements of the acute psychedelic experience (measured via the EBI, MEQ and CEQ) and long-term psychological effects (well-being, measured via the WEMWBS), in line with our prior aim that the PIS be a useful subacute predictor or mediator of long-term psychological changes. To demonstrate convergent validity, the relationship between PIS scores and previously validated insight scales was analysed.

The PIS appears to be a sensitive scale, yielding high and rangeful scores in both of the studies in which it was deployed. In terms of predictive and concurrent validity, our two corresponding hypotheses were confirmed: that is, PIS measured insight 1 day post-experience was predictive of well-being 2 weeks post-retreat (H1), with the lower baseline well-being subgroup demonstrating a greater correlation than the total sample. PIS measured insight at 2 weeks post-retreat demonstrated a positive relationship with well-being at the same timepoint (H2). Regarding our hypothesis on the postdictive validity of the PIS (H3), emotional breakthrough predicted insight at 2 weeks after the psychedelic experience, as did mystical experience in study 2 (but not study 1). Challenging experiences failed to predict insight in either study. In study 1, PIS scores at +1 day appeared to mediate the relationship between emotional breakthrough and well-being after 2 weeks (H4); however, psychological insight did not mediate the effects of mystical experiences on well-being.

The main purpose of study 2 was to assess the external validity of these findings.^1^ Results from these analyses largely confirmed our initial findings, providing additional support for our prior hypotheses that PIS scores 1 day post-psychedelic experience would predict subsequent changes in well-being 2 weeks later, and that PIS scores at 2 weeks would correlate with well-being changes (from baseline) sampled at the same timepoint. Results from study 2 support the view that even in a non-specific setting, psychedelics maintain the capacity to facilitate psychological insight. There was some suggestion of a different temporal profile to PIS scores in studies 1 and 2, with a slight incremental decrease in scores over time in study 1 but not in study 2, where a somewhat opposite profile was apparent. However, no formal analyses were applied in this regard. It is tempting to speculate about the impact of the different contexts of use on the respective temporal profiles of PIS scores in study 1 versus study 2, for example, greater support 1 day post-experience amplifying early insight scores in study 1 (the ceremony study), but greater experimental control would be needed to properly assess such a hypothesis.

The convergent validity of the PIS was demonstrated in study 2 by comparing scores with existing subjective measures of insight. A weak convergence between SRIS-IN and PIS scores indicates that the PIS assesses a somewhat distinct construct. Items in the SRIS appear to explore trait as opposed to experience-referenced insight: for example, ‘I usually know why I feel the way I do’, ‘I usually have a very clear idea about why I’ve behaved in a certain way’ (Grant et al., 2002). In contrast, the MIS showed highly convergent scores with those from the PIS. The MIS was originally devised to examine insights following a therapy session with items, including ‘I feel I’ve got more understanding of reasons behind my behaviour and feelings’ and ‘I feel I’ve got ideas for better ways of dealing with people and problems’. Thus, like the PIS, the MIS explores how insight has changed with respect to a specific ‘event’ or process (Kolden, 1991). Note, however, that the PIS can also be used to measure insight referenced to non-psychedelic drug-induced experiences, and this is made clear in its wording (see appendices of Supplemental Material). Similarly, the MIS has been adapted for use in relation to the discussion of dream content and at its interpretation (Kolden et al., 2000).

### Insight and emotional breakthroughs

Psychodynamic therapy (Fisher et al., 2016) and modern, third-wave psychotherapies such as mindfulness-based CBT and acceptance and commitment therapy (ACT) encourage emotional exploration and insight (for a review, see Grosse Holtforth et al., 2007). It has been shown (Johansson et al., 2010) and argued (Fosha, 2009) that insight associated with intense emotion promotes more enduring, therapeutically beneficial effects from psychotherapy (Fosha, 2009; Greenberg and Safran, 1987). In addition, insight has also been shown to be a positive mediator of change in psychodynamic therapy (Johansson et al., 2010). In this context, the fact that our results indicate an insight-mediated relationship between emotional breakthroughs and well-being is promising.

A major focus of the EBI is an individual’s ability to face emotionally difficult feelings or past experiences and, in so doing, experience emotional breakthrough, which overlaps somewhat with our operational definition of insight, as indexed by the PIS. For context, please see examples of the EBI items below (Roseman et al., 2019):

*I faced emotionally difficult feelings that I usually push aside.*

*I felt able to explore challenging emotions and memories.*

*I was able to get a sense of closure on an emotional problem.*

And following are some items from the PIS:

*I have learned important new ways of thinking about my ‘self’ and my problems.*

*I have become more conscious of aspects of my past that I used to ignore or not be fully aware of.*

Hence, at least as far as is suggested by the scales we have devised, emotional breakthrough and psychological insight are interrelated. Indeed, prior to their construction, there was an assumption that the type of insight captured via the PIS often follows the overcoming of some psychological resistance (Roseman et al., 2019). Thus, the EBI refers to a dynamic, affective process (which presumably has a specific neurobiology) occurring during a psychedelic experience, whereas the PIS indexes a more reflective, cognitive or epistemic processing taking place after the psychedelic experience – and hence after any related emotional breakthrough. We are mindful, however, that this temporal distinction between the EBI and PIS may not apply absolutely, for example, emotional breakthroughs may occur after a psychedelic experience, and experiences of psychological insight may occur during it. Moreover, the relationship between acute emotional breakthrough (or acute insight) and subsequent post-acute insight may be continuous rather than discrete.

One way to observe the co-occurrence of acute insights and emotional breakthroughs would be to utilise current measures of acute insightful experiences alongside the EBI. In a recently published paper, *acute* insightful experiences are measured using a novel scale, the PIQ (Davis et al., 2021b). There are some similarities between the two scales (e.g. from the EBI: ‘I faced emotionally difficult feelings that I usually push aside’ and ‘I felt able to explore challenging emotions and memories’ vs the following from the PIQ: ‘Awareness of uncomfortable or painful feelings I previously avoided’ and ‘[I] Realized I could experience memories previously too difficult to experience’). There are large differences, however, between the two scales: the PIQ appears to examine transpersonal or philosophical insights, and awareness of one’s perspectives, past events and beliefs, whereas the EBI explores the overcoming of psychological resistance potentiating relief, breakthrough and/or resolution. The PIQ and EBI would complement each other, enabling cross-examination of acute insights and emotional breakthroughs in future psychedelic studies. Utilising both scales alongside the PIS would broaden our awareness of the phenomenology behind psychedelic experiences.

It is important to note that the PIS does not take into account whether perceived insights have a positive or negative emotional valence. Enhanced psychological insight may intuitively appear to have an adaptive function, with empirical evidence supporting this (Harrington and Loffredo, 2011; Lyke, 2009). However, some previous work also suggests that for individuals who have negative self-perceptions, greater self-insight can be associated with greater depressive symptoms (Nakajima et al., 2018; Stein and Grant, 2014). Moreover, some studies suggest that individuals with depression, anxiety or schizophrenia, who show greater insight into their condition, that is, they have ‘clinical insight’, also have more severe depressive symptomatology (Sanz et al., 1998). The literature on this topic is somewhat conflicting, however (de Assis da Silva et al., 2017; Murri et al., 2015; Gorwood et al., 2019), which could be explained by differing definitions of insight. While results in the present study indicate that psychological insight, as measured by the PIS, is positively associated with mental well-being, future studies could explore how psychedelic facilitated psychological and clinical insights are associated with psychological outcomes in cohorts with various psychopathologies. The role of destabilisation in the psychotherapeutic process may be relevant here (Olthof et al., 2020).

### Insight, mystical experiences and challenging experiences

Here, the extent of psychedelic occasioned mystical experiences, as measured via MEQ, was not reliably predictive of psychological insight at +2 weeks across studies 1 and 2. Moreover, study 1 results show that psychological insight did not significantly mediate the effect of mystical experiences on well-being. Our definition of psychological insight places greater focus on subjective personal insight bearing relevance to one’s own self and life, as opposed to insight of a transpersonal nature, related to such things as the nature of consciousness, life and existence, which may partially explain the present findings. In the same way that the present study found a relationship between emotional breakthrough and psychological insight (of the personal type), there may be a parallel relationship between unitive experiences (e.g. as indexed by the MEQ) and this more transpersonal, philosophical type of insight. This hypothesis could be tested in future studies by using either existing (Hanley et al., 2018; MacLean et al., 2012) or new measures of the unitive experience, as well as a new measure of transpersonal insight or shifting high-level (e.g. metaphysical) beliefs or perspectives.

With regard to challenging psychedelic experiences, previous studies have demonstrated mixed results for the relationship between challenging experiences and long-term psychological outcomes (Barrett et al., 2016; Carbonaro et al., 2016; Haijen et al., 2018) and here we found minimal interaction between CEQ and PIS scores, as well as CEQ and well-being. The CEQ is a multifaceted scale, containing items pertaining to a range of psychological phenomena. For example, the subscale ‘Physical Distress’ includes items such as ‘Feel heart beating’ and has been found to be associated with decreases in well-being (Barrett et al., 2016). Conversely, the CEQ also contains a ‘Grief’ subscale. Grief has previously been shown to be part of the process of emotional recovery after trauma (Bonanno, 2001). The CEQ also encompasses items that probe psychotomimetic symptoms, including paranoia and delusions (e.g. ‘I had the feeling that people were plotting against me’ and ‘Fear that I might lose my mind or go insane’). The multifaceted nature of the CEQ may explain why studies have reported mixed results for the relationship between challenging experiences and long-term psychological outcomes (Barrett et al., 2016; Carbonaro et al., 2016; Haijen et al., 2018).

### Insight and well-being

In line with evidence supporting the importance of insight in the psychotherapeutic process, we found that psychological insight was more strongly predictive of changes in well-being in a low well-being subgroup (Figures 2 and 3). This supports previously discussed evidence that psychedelics may hold more potential for those with low levels of well-being, which often occurs in mental health disorders, and that insight is interlinked with this potential improvement in well-being.

While we found evidence that personal psychological insight mediates the relationship between emotional breakthrough and improved well-being, such insight may be a significant contributor to improved mental well-being in and of itself. Indeed, in the present study, we found evidence to support the 1-2-1 relationship between insight and well-being (H2). In previous studies, high levels of psychological insight have been shown to be related to improved well-being and overall life satisfaction (Harrington and Loffredo, 2011; Lyke, 2009), correlating with decreases in scores relating to depression (Silvia and Phillips, 2011) and anxiety (Grant et al., 2002). Moreover, previous research has postulated that psychedelic-induced insight is a key factor in the acute psychological effects that ultimately lead to a change in addictive behaviour (Bogenschutz and Pommy, 2012). This was supported by later findings demonstrating that patients gained profound insights into their self-identity, leading to smoking cessation (Noorani et al., 2018). Relatedly, following psilocybin-assisted therapy for individuals with major depressive disorder (MDD), significant reductions in depression were associated with more accurate forecasting of negative future events (Lyons and Carhart-Harris, 2018). This phenomenon could relate to psychological insight – as by having more awareness and insight into one’s maladaptive thought patterns, one may have greater capacity to evaluate and adjust previously aberrant negative biases, thus enabling more accurate forecasting of one’s future. Within other clinical populations, deficits in insight and self-awareness have been documented and studied in patients with obsessive compulsive disorder (OCD) (Marazziti et al., 2012). Specifically, in patients with OCD, lower self-reflection or insight into their condition has been associated with greater severity of OCD symptoms, psychiatric comorbidities (Bellino et al., 2005) and poorer response to treatment (Catapano et al., 2010). These findings may help explain why psilocybin has shown some preliminary signs of efficacy for patients with this disorder (Moreno et al., 2006; Wilcox, 2014).

To build on these findings, future studies may look to assess the hypothesis that insight mediates the relationship between emotional breakthroughs and well-being to an even greater extent in clinical populations, and assess whether greater psychological integration can enhance this effect. Moreover, given that the PIS was conceived as a scale that could be used for experiences other than just psychedelic experiences, future studies could seek to employ the scale in relation to other, non-psychedelic events or processes to further assess its validity and usefulness. Some examples might include major geographical journeys, life events or series of them, undergoing a course of psychotherapy or even a conventional course of a specific psychiatric intervention such as electroconvulsive therapy. Assessed in this way, we would predict that psychedelic experiences have an especially potent impact on psychological insight and that epistemic development is a core component of the psychedelic-therapy treatment model.

### Limitations

When interpreting the present results, one should be cognisant of the subjective nature of insight, particularly when it is sampled using subjective rating scales. The matter of the accuracy of alleged insight in relation to psychedelic experiences has previously been the topic of an extensive review paper (Letheby, 2016). What the experiencer chooses to do with his or her self-perceived insight, and how it is dealt with therapeutically, is a rich topic for future research and debate (Timmermann et al., 2020). Moreover, considering the psychedelic state can entail transient psychotomimetic symptoms including delusional beliefs and paranoid thinking (Johnstad, 2021), future studies might explore whether any persisting delusions are being misinterpreted as insights.

This present study utilised opportunistic sampling and therefore its populations were a mixture of psychologically healthy and unwell individuals: for example, 280 out of 886 participants reported having some lifetime diagnosis of at least one psychiatric illness in study 1, and 91 of 279 reported this in study 2. This was one of the reasons we decided to run subanalyses on a low well-being subgroup. Future analyses might look to treat these populations as separate, for example, to assess potential differences in PIS scores and their relationships with other salient outcomes, such as those pertaining to mental health.

In terms of the demographics of the study populations, in study 2, males made up 58% of the sample and Caucasians, 78%. These values may be representative of the psychedelic using population in similar studies (Davis et al., 2021b), but it is not clear whether they are representative of the general populations from which they were drawn. If not, greater efforts may be needed to ensure fuller representation. The majority of participants within both studies had prior psychedelic experience (60% and 81%, studies 1 and 2, respectively) and baseline assessment revealed some potential for a positive perspective bias towards psychedelics. Expectation scores could be entered as a covariate in future analyses to account for this potential driver of positive outcomes. For study 1, 89.8% of participants reported use of classic psychedelics specifically and this proportion was very high (96%) for study 2. An aim of the present study was to explore insight following general psychedelic use; therefore, outcomes cannot be exclusively related to use of classic psychedelics only. Future studies could assess drug-dependent relationships for the PIS, including the question of whether psychological insight is a specific property of classic psychedelic drug experiences.

Greater efforts should be made in future studies to both minimise attrition rates and assess what factors are contributing to them, as done here (Hübner et al., 2021). Fortunately, initial explorations of potential attrition bias in the present datasets have generally been reassuring. The observational nature of these studies limits our ability to obtain accurate and verifiable information on dosage, timing of dosing and type of drug used. This is standard drawback of observational studies. Future work could look to repeat similar analyses but from data derived from controlled studies, something we are presently working on. Given that the PIS is a retrospective measure and not a prospective one (i.e. ‘pre vs post’), a controlled study with inactive (e.g. a placebo) or active drug controls will enable us to gain a much better impression of the *specificity* of psychedelic-experience induced insight.

Within the current study, a panel of experts reviewed the content of the scale and qualitative evidence of insight was assessed. However, the present study did not include a formal measure of content validity, for example, calculation of a validity index. A mixed methods approach such as combined independent ratings as well as quantitative and qualitative evidence (Benz and Newman, 2008) would ensure a broader validation process of scale content.

Increased well-being is one of the most reliable psychological changes following a psychedelic experience and relevant measures of well-being such as the WEMWBS, offer a generic index of mental health. There are, however, many components to mental health (Feldman et al., 2007). It would be an extension to the present work to measure the relationship between insight and some other psychological domains, such as resilience, experiential avoidance, specific symptom domains or domains of personality known to be sensitive to change post-psychedelics (Erritzoe et al., 2018; MacLean et al., 2011).

The present study’s findings highlight potential mental health benefits from psychedelic use. However, it is important not to neglect anomalous cases where negative health outcomes have occurred after psychedelic use, even if these cases are rare outliers in a dataset. It is important to note that precautionary steps within modern psychedelic trials ensure patient safety (both psychological and physiological), for example, by screening personal and family history of psychotic conditions, promoting a safe environment, ensuring rapport is built between the session psychologists and participants and providing psychological support prior to and throughout the dosing session as well as in the days/weeks following the experience. These guidelines for safety are now adopted as standard best practice for human psychedelic research and may have inspired procedures in the ceremonies from which our study 1 data are derived (Johnson et al., 2008). In future studies, we plan to focus on anomalous negative psychological responses to psychedelics with the aim of better understanding their nature – including *how*, *why* and *when* they occur, in order to further mitigate their risk of occurrence.

## Conclusion

We have introduced a new scale, the PIS, intended to sample personal psychological insight emerging after a psychedelic experience. We anticipate that the PIS will be a useful addition to the arsenal of subjective measures used by contemporary and future psychedelic researchers – and may also inspire newer, improved scales. The present study has demonstrated that psychological insight plays an important role in mediating positive psychological outcomes after a psychedelic experience, particularly through its relationship with emotional breakthrough, where it mediates the relationship between emotional breakthrough and subsequent improvements in psychological well-being. The validation of the PIS now opens up the exciting prospect of better studying the neurobiology of insight and insight-related processes using this and related measures twinned with functional brain imaging.

## Supplemental Material

sj-docx-1-jop-10.1177_02698811211066709 – Supplemental material for Validation of the Psychological Insight Scale: A new scale to assess psychological insight following a psychedelic experience Click here for additional data file. Supplemental material, sj-docx-1-jop-10.1177_02698811211066709 for Validation of the Psychological Insight Scale: A new scale to assess psychological insight following a psychedelic experience by Joseph M Peill, Katie E Trinci, Hannes Kettner, Lea J Mertens, Leor Roseman, Christopher Timmermann, Fernando E Rosas, Taylor Lyons and Robin L Carhart-Harris in Journal of Psychopharmacology

## Notes

1. Note that the same exact analyses were not utilised for study 1 and 2 due to the differences in the temporal structure of the surveys, thus mediating effect of insight was not explored in relation to the global psychedelic survey data.
