# Supplementary material for: Validation of the Psychological Insight Scale: A new scale to assess psychological insight following a psychedelic experience
Source: J Psychopharmacol. 2022 Jan 5;36(1):31–45. doi: 10.1177/02698811211066709 (PMC8801624; doi:10.1177/02698811211066709)
Supplement: sj-docx-1-jop-10.1177_02698811211066709 – Supplemental material for Validation of the Psychological Insight Scale: A new scale to assess psychological insight following a psychedelic experience [file sj-docx-1-jop-10.1177_02698811211066709.docx]

**Appendices (Words = 561)**

*The psychological insight scale (PIS)*

*Description.* Please rate each item in relation to whether you have noticed any change in your level of insight compared with your **usual/baseline state**, e.g. **before beginning a particular treatment** and/or experience that this scale may be assessing. Please rate according to whatever the duration of time has been since this particular treatment/experience began.

| **Table 4.** Items of the PIS | |
| --- | --- |
| Item | Question |
| 1 | *I have had important new insights about how past events have influenced my current mental health and behaviour* |
| 2 | *I have learned important new ways of thinking about my ‘self’ and my problems* |
| 3 | *I have had important new insights about how I would like to change aspects of myself or my lifestyle* |
| 4 | *I have become more conscious of aspects of my past that I used to ignore or not be fully aware of* |
| 5 | *I have become more conscious of aspects of my ‘self’ that I used to ignore or not be fully aware of* |
| 6 | *I have become more conscious of aspects of my lifestyle than I used to ignore or not be fully aware of* |

No more than at baseline

Much more than at baseline

Please rate the following item in the same way as the previous 6, i.e. in relation to whether you have noticed a change in your current state relative to **before beginning** the relevant treatment and/or experience that this scale may be assessing:

1. I have made **positive changes** to my lifestyle and/or behaviour in accordance with the insights I have gained as part of my treatment/experience.

I have made no noticeable positive changes to my lifestyle and/or behaviour

I have made significant positive changes to my lifestyle and/or behaviour

*Scoring.* Total PIS scores are calculated as the mean of the ratings for items 1- 6. Item 7 is separate as it gives an index of behavioural change in association with potential insights. Scores range from 0-100.

| **Table 5.** Direct, indirect and total effects of the path analysis predicting post-acute insight and psychological well-being two weeks after a psychedelic experience, based on the acute psychedelic state | | | | | | | |
| --- | --- | --- | --- | --- | --- | --- | --- |
|  |  | |  | | 95% Confidence Interval | |  |
|  | Unstandardized Estimate B | Standard Error (SE) | z | *p*-value | Lower limit | Upper limit | Standardized estimate β |
| **Direct effects:** |  |  |  |  |  |  |  |
| WB2 |  |  |  |  |  |  |  |
| ~ CEQ | -0.048 | 0.019 | -2.55 | .01 | -0.085 | -0.011 | -0.121 |
| ~ MEQ | 0.053 | 0.013 | 3.92 | <.0001 | 0.026 | 0.079 | 0.235 |
| ~ EBI | 0.005 | 0.003 | 1.37 | .17 | -0.002 | 0.011 | 0.095 |
| ~ PIS | 0.014 | 0.004 | 3.78 | <.001 | 0.007 | 0.021 | 0.222 |
| ~ WB1 | 0.427 | 0.048 | 8.82 | <.0001 | 0.332 | 0.522 | 0.408 |
| PIS |  |  |  | - |  |  |  |
| ~ CEQ | 0.447 | 0.294 | 1.52 | .13 | -0.13 | 1.024 | 0.072 |
| ~ MEQ | 0.321 | 0.209 | 1.53 | .13 | -0.09 | 0.731 | 0.091 |
| ~ EBI | 0.436 | 0.047 | 9.21 | <.0001 | 0.343 | 0.528 | 0.566 |
| **Indirect effects:** |  |  |  |  |  |  |  |
| WB2 |  |  |  |  |  |  |  |
| ~ CEQ * PIS | 0.006 | 0.004 | 1.41 | .16 | -0.002 | 0.015 | 0.016 |
| ~ MEQ * PIS | 0.004 | 0.003 | 1.42 | .16 | -0.002 | 0.011 | 0.02 |
| ~ EBI * PIS | 0.006 | 0.002 | 3.50 | <.001 | 0.003 | 0.01 | 0.126 |
| **Total effects** |  |  |  |  |  |  |  |
| WB2 |  |  |  |  |  |  |  |
| ~ CEQ + (CEQ * PIS) | -0.042 | 0.019 | -2.17 | .03 | -0.08 | -0.004 | -0.105 |
| ~ MEQ + (MEQ * PIS) | 0.057 | 0.014 | 4.17 | <.0001 | 0.03 | 0.084 | 0.255 |
| ~ EBI + (EBI * PIS) | 0.011 | 0.003 | 3.54 | <.001 | 0.005 | 0.017 | 0.221 |
| *Note. CEQ: Challenging Experience Questionnaire; MEQ: Mystical Experience Questionnaire; EBI: Emotional Breakthrough Inventory; PIS: Psychological Insight Scale; WB1, WB2: Psychological well-being at baseline and 2 weeks post-retreat, respectively, measured with the Warwick-Edinburgh Mental Well-being Scale (WEMWBS).* | | | | | | | |
